# Macular Ganglion Cell Complex and Peripapillary Retinal Nerve Fibre Layer Changes in Patients With Thyroid Eye Disease Without Optic Neuropathy: A Retrospective Observational Study

**DOI:** 10.7759/cureus.91168

**Published:** 2025-08-28

**Authors:** Shrinkhal, Aparajita Shukla, Pragati Garg, Nilakshi Banerjee, Pramod Kumar, Mukesh Shukla, Ruchi Shukla, Swarastra P Singh

**Affiliations:** 1 Ophthalmology, All India Institute of Medical Sciences, Raebareli, Raebareli, IND; 2 General Medicine, All India Institute of Medical Sciences, Raebareli, Raebareli, IND; 3 Community and Family Medicine, All India Institute of Medical Sciences, Raebareli, Raebareli, IND

**Keywords:** macular ganglion cell complex, peri-papillary retinal nerve fibre layer, retinal nerve fibre layer (rnfl), ted, thyroid eye disease

## Abstract

Background: Thyroid eye disease (TED) is an autoimmune inflammatory disorder affecting orbital tissues and can lead to optic nerve damage. Early detection of retinal changes before clinical signs of optic neuropathy is crucial to prevent vision loss.

Objective: To evaluate macular ganglion cell complex (GCC) and peripapillary retinal nerve fiber layer (RNFL) thickness in patients with TED without clinical signs of optic neuropathy.

Methods: A retrospective observational study was conducted at All India Institute of Medical Sciences, Raebareli, India, between January 2024 and January 2025. Patients with TED and best-corrected visual acuity (BCVA) of 6/9 or better were included. Based on intraocular pressure (IOP), gender, and age, patients were categorized to assess their association with structural retinal parameters. Spectral-domain optical coherence tomography (SD-OCT) was used to evaluate GCC and RNFL thickness.

Results: The mean age of 50 patients enrolled in the study was 43.03 ± 1.79 years. The majority (84%) of the patients were females. Although not statistically significant, patients with elevated IOP had lower RNFL and GCC-inner plexiform layer (GCL-IPL) thickness. Male patients exhibited significantly higher GCL-IPL thickness in the inferotemporal and superotemporal sectors. Age was negatively correlated with GCL-IPL thickness, particularly in the inferotemporal and superotemporal regions.

Conclusion: TED patients with elevated IOP, increasing age, and female gender exhibited thinner RNFL and GCL-IPL, though most differences were not statistically significant. These findings underscore the importance of early OCT monitoring in TED patients to detect subclinical optic nerve changes. Future longitudinal studies with larger sample sizes and appropriate control groups are needed to confirm these associations and to better establish causality.

## Introduction

Thyroid eye disease (TED) is the leading orbital disorder, affecting approximately 0.25-0.5% of the general population, with an incidence of 16 per 100,000 females and 2.9 per 100,000 males, and shows no significant ethnic variation [1]. It is most commonly associated with Graves’ disease, occurring in about 25-50% of affected patients, and can present with a spectrum of manifestations ranging from mild eyelid retraction to severe orbital inflammation, proptosis, and vision-threatening complications [2]. TED is an autoimmune condition in which the immune system mistakenly targets orbital tissues, leading to inflammation and swelling [3]. More specifically, TED involves the autoimmune attack on orbital fibroblasts expressing the thyroid-stimulating hormone receptor (TSHR) and insulin-like growth factor-1 receptor (IGF-1R), leading to inflammation, adipogenesis, and fibrosis. Excess cytokine production, including interleukin-1 and prostaglandins, accelerates tissue damage and disease progression [4].

The development of TED also has a strong genetic basis, with cytotoxic T-lymphocyte-associated protein 4 (CTLA-4), human leukocyte antigen DR beta 1 (HLA-DRB1), and tumor necrosis factor-alpha (TNF-α) genes being commonly implicated [5]. Excessive hyaluronic acid production causes orbital expansion and fibrosis, manifesting clinically as soft tissue inflammation, proptosis, and ophthalmoplegia - features collectively known as Graves’ ophthalmopathy or TED [6]. TED is driven by an intricate immune-mediated loop involving orbital fibroblasts, adipocytes, and lymphocytes. This continuous cycle of immune activation, fibroblast proliferation, and extracellular matrix remodeling results in the characteristic orbital changes seen in TED [7].

Pathologically, TED involves an increase in orbital tissue volume due to mucopolysaccharide infiltration, which elevates intraorbital pressure. In severe cases, this can lead to optic nerve compression, ischemia, and vision loss. TED is often associated with other autoimmune diseases such as rheumatoid arthritis, Addison’s disease, and systemic lupus erythematosus, which increase susceptibility [8].

Environmental factors, especially smoking, also play a pivotal role. Smoking worsens TED by increasing oxidative stress, immune dysregulation, and orbital inflammation, while simultaneously reducing treatment responsiveness. Smoking cessation is thus critical for slowing disease progression and improving therapeutic outcomes [9]. TED occurs in approximately 90% of hyperthyroid cases, primarily due to Graves’ disease, but can also affect 6% of euthyroid individuals, 3% of those with Hashimoto’s thyroiditis, and 1% of those with primary hypothyroidism, indicating a complex autoimmune mechanism independent of thyroid hormone levels [2].

TED demonstrates ethnic variability. African American individuals face the highest risk and more severe manifestations, such as pronounced proptosis and optic neuropathy, followed by White populations. Asians have a lower prevalence but may exhibit more restrictive strabismus. These variations may result from genetic, environmental, and immune differences [10]. TED also exhibits a bimodal age distribution: 40-44 and 60-64 years in females, and 45-49 and 65-69 years in males. Hormonal and immunologic factors may influence age-specific vulnerability [2]. Older patients are particularly prone to severe manifestations such as restrictive myopathy and dysthyroid optic neuropathy (DON) [7], and 3-7% of patients experience vision-threatening DON [11].

While TED is more prevalent in females due to higher autoimmune susceptibility, males typically exhibit more severe ocular involvement and poorer visual outcomes [12]. Psychological stress exacerbates TED by activating the hypothalamic-pituitary-adrenal (HPA) axis and promoting cytokine-mediated immune responses. Chronic corticosteroid use may further dysregulate immunity, aggravating disease severity [13]. Pregnancy, particularly the postpartum period, increases the risk of TED by up to 30% in women with Graves’ disease, likely due to immune rebound and hormonal changes [14]. Other contributing risk factors include trauma, which may trigger orbital autoimmunity, and elevated serum cholesterol, which has been associated with disease progression [15].

Graves’ disease is also linked to increased IOP, raising the risk of glaucomatous optic nerve damage. Orbital congestion, elevated episcleral venous pressure, and impaired aqueous outflow contribute to this pathogenesis. Chronic inflammation and tissue expansion further predispose to glaucomatous neuropathy and visual decline [16].

Although clinically evident DON occurs in only 3-7% of patients, subclinical optic neuropathy may be present even in mild TED. Optical coherence tomography (OCT) is a valuable tool for detecting early structural changes in the retina and optic nerve. Studies have shown that retinal nerve fiber layer (RNFL) thinning in TED can precede overt signs of optic neuropathy. Analysis of the ganglion cell complex (GCC) has emerged as a more sensitive marker for early optic nerve involvement.

Danesh-Meyer et al. [17] observed significant RNFL changes in patients with chiasmal compression, suggesting that compressive optic neuropathy affects the RNFL. Bartalena et al. [18] demonstrated that subclinical optic neuropathy can occur in TED patients with only mild soft tissue involvement, as evidenced by reduced RNFL thickness on OCT. Mugdha et al. [19] further showed persistent RNFL thinning in TED even in the absence of active disease, indicating underlying compressive optic damage. RGC layer measurements may be more sensitive than RNFL analysis alone, making them essential in the early detection and monitoring of optic neuropathies [20].

Although TED is often bilateral, it can present asymmetrically, with one eye demonstrating more severe structural or functional damage than the other. Therefore, eye-specific analysis was performed to capture potential inter-eye differences that may serve as early clinical indicators of disease involvement.

This study aims to evaluate RNFL and GCC changes in TED patients without clinical evidence of DON, focusing on parameters such as intraocular pressure (IOP), gender, and age. By identifying early structural alterations, this study hopes to support early diagnosis and timely intervention, which could potentially prevent disease progression and vision loss. A better understanding of these changes may also offer insights into the subclinical effects of TED on retinal and optic nerve health.

## Materials and methods

Following approval by the Institutional Ethics Committee, a retrospective observational study was conducted in the Department of Ophthalmology at All India Institute of Medical Sciences, Raebareli, Raebareli, India, in collaboration with the Department of General Medicine. Patients diagnosed with TED between January 2024 and January 2025 were included.

Patient data were extracted from the hospital information system (HIS) and OCT machine records. Inclusion criteria included a best-corrected visual acuity (BCVA) of 6/9 or better and clinical features of TED. TED was defined by the presence of eyelid retraction, characterized as the upper lid margin being at or above the superior limbus or the lower lid margin positioned below the inferior limbus in primary gaze without frontalis overaction, along with at least one of the following: exophthalmos greater than 18 mm as measured by Hertel’s exophthalmometer; extraocular muscle involvement based on clinical examination or radiological evidence from ultrasound, computed tomography (CT), or magnetic resonance imaging (MRI); biochemical thyroid dysfunction including either hyperthyroidism or hypothyroidism; or a Clinical Activity Score (CAS) greater than 3 out of 7.

Exclusion criteria included BCVA worse than 6/9, clinical signs of optic nerve dysfunction (abnormal pupillary reactions, color vision defects, or visual field loss), other retinal or optic nerve-affecting ocular conditions (e.g., diabetic or hypertensive retinopathy), IOP >18 mmHg (patients with baseline IOP >18 mmHg at recruitment were excluded; however, relative or episodic elevations during follow-up (mean values around 18.6 mmHg) were reported separately in the results to reflect intra-study variation), or OCT scans with signal strength <5/10.

In this study, subclinical optic neuropathy was defined as structural or functional changes detected on OCT or visual field testing in the absence of overt clinical signs such as relative afferent pupillary defect (RAPD), dyschromatopsia, or field loss.

All patients underwent detailed ophthalmic evaluation, including history-taking (thyroid status, treatments, systemic comorbidities), BCVA assessment using the Snellen chart, slit-lamp biomicroscopy, and IOP measurement using Goldmann applanation tonometry.

Spectral-domain optical coherence tomography (SD-OCT; Cirrus HD-OCT 5000, model 5000-24512; version 11.5.2.54532, Carl Zeiss Meditec, Inc., Dublin, CA, USA) was used to obtain single, high-quality scans of each eye. All scans were reviewed for centration, segmentation accuracy, and signal strength, with a minimum acceptable signal strength score of 5 out of 10. Measurements included ganglion cell complex-inner plexiform layer (GCC-IPL) thickness, comprising both average and sectoral values (superior, superior nasal, inferior nasal, inferior, inferotemporal, and superotemporal), and RNFL thickness, assessed as the average value using a 360° peripapillary scan with a 3.46 mm diameter. A central 6 mm macular scan was used for GCC analysis. Patients were stratified based on IOP status (normal vs. elevated), gender (male vs. female), and age group. Statistical analysis was performed to evaluate variations in GCC-IPL and RNFL thickness across these subgroups.

Statistical analysis

All statistical analyses were performed using IBM SPSS Statistics for Windows, Version 27 (Released 2021; IBM Corp., Armonk, New York, United States). Continuous variables were expressed as mean ± standard deviation (SD) or median with interquartile range (IQR), as appropriate. The Mann-Whitney U test was used to compare RNFL and GCL-IPL thickness between groups based on IOP and gender, due to the non-parametric distribution of the data. Spearman’s correlation coefficient was employed to assess associations between age and retinal thickness parameters. A p-value < 0.05 was considered statistically significant.

## Results

A total of 50 participants were included in the study. The mean age was 43.03 ± 1.79 years, with a median age of 44.0 years (IQR: 33-44 years). The cohort comprised predominantly female patients (84.0%), with males representing 16.0% of the study population.

Intraocular pressure (IOP)

Raised IOP was observed in 20.0% of left eyes and 28.0% of right eyes. In the left eye, the mean IOP was 18.60 ± 3.31 mmHg (median: 18.1 mmHg; IQR: 16.37-18.10 mmHg). In the right eye, the mean IOP was 18.30 ± 3.14 mmHg (median: 18.1 mmHg; IQR: 16.05-18.10 mmHg) (Table 1).

**Table 1:** Sociodemographic and Clinical Profile of Study Participants IOP: intraocular pressure; IQR: interquartile range; SD: standard deviation

Study Variable	Number (%)
Age (years)	
<30	7 (14.0)
31-45	20 (40.0)
46-60	17 (34.0)
>60	6 (12.0)
Mean ± SD (median; IQR)	43.03 ± 1.79 (44.0; 33-44)
Gender	
Male	8 (16.0)
Female	42 (84.0)
IOP	
Left eye	
Raised	10 (20.0)
Normal	40 (80.0)
Mean ± SD (N = 50)	18.60 ± 3.31 (18.1; 16.37-18.10)
Right eye	
Raised	14 (28.0)
Normal	36 (72.0)
Mean ± SD (N = 50)	18.30 ± 3.14 (18.1; 16.05-18.10)

RNFL and GCL-IPL thickness according to IOP

The average RNFL and GCL-IPL thicknesses were generally lower in patients with raised IOP compared to those with normal IOP, in both eyes. However, these differences did not reach statistical significance.

In the right eye, the median RNFL thickness in the raised IOP group was 89.0 µm (IQR: 81.75-93.50), compared to 88.0 µm (IQR: 87.00-96.00) in the normal IOP group (p = 0.82). In the left eye, the median RNFL thickness was 89.5 µm (IQR: 83.25-95.00) in the raised IOP group versus 88.8 µm (IQR: 83.25-94.75) in the normal IOP group (p = 0.93).

Similarly, GCL-IPL thicknesses were lower in eyes with elevated IOP. For the right eye, the median GCL-IPL thickness was 80.0 µm (IQR: 71.00-87.50) in the raised IOP group versus 81.5 µm (IQR: 77.00-85.75) in the normal IOP group (p = 0.58). For the left eye, the values were 80.0 µm (IQR: 67.00-83.75) and 80.0 µm (IQR: 74.75-84.00), respectively (p = 0.54).

This trend of lower GCL-IPL thickness in the raised IOP group was observed across all sectors. In the right eye, superior GCL-IPL thickness was 80.5 µm (IQR: 72.25-85.50) in the raised IOP group versus 80.0 µm (IQR: 75.25-86.50) in the normal IOP group (p = 0.37). In the left eye, the superior GCL-IPL thickness was 78.5 µm (IQR: 65.75-81.50) in the raised IOP group and 80.5 µm (IQR: 73.75-85.00) in the normal IOP group (p = 0.52).

Other GCL-IPL sectoral values (superior nasal, inferior nasal, inferior, inferotemporal, and superotemporal) also showed slight reductions in the raised IOP group, though none of these differences were statistically significant (p > 0.05 for all comparisons).

Overall, while patients with elevated IOP exhibited lower RNFL and GCL-IPL thicknesses, these findings were not statistically significant (Table 2).

**Table 2:** Comparison of RNFL and GCL-IPL Thickness in Eyes Based on IOP Status (N = 50) All values are reported as median (IQR). Statistical test: Mann-Whitney U test; p < 0.05 considered statistically significant. RE: right eye; LE: left eye; RNFL: retinal nerve fiber layer; GCL-IPL: ganglion cell layer-inner plexiform layer; IOP: intraocular pressure; AVG: average; IQR: interquartile range

Nerve Parameter	Raised IOP RE (N=14)	Normal IOP RE (N=36)	P-value (RE)	Raised IOP LE (N=10)	Normal IOP LE (N=40)	P-value (LE)
AVG RNFL	89.0 (81.75-93.50)	88.0 (87.00-96.00)	0.82	89.5 (83.25-95.00)	88.8 (83.25-94.75)	0.93
AVG GCL-IPL	80.0 (71.00-87.50)	81.5 (77.00-85.75)	0.58	80.0 (67.00-83.75)	80.0 (74.75-84.00)	0.54
GCL-IPL superior	80.5 (72.75-85.75)	82.0 (75.25-87.75)	0.37	80.5 (63.25-84.25)	80.0 (76.00-85.00)	0.52
GCL-IPL superior nasal	82.5 (71.00-88.25)	83.5 (77.00-88.00)	0.50	81.0 (71.75-84.25)	81.5 (76.00-86.00)	0.29
GCL-IPL inferonasal	82.5 (71.00-88.25)	81.0 (74.00-87.00)	0.84	76.0 (71.75-82.50)	80.5 (76.25-85.75)	0.11
GCL-IPL inferior	79.5 (72.25-87.25)	79.5 (74.00-85.00)	0.75	74.0 (61.25-83.75)	79.0 (73.25-84.00)	0.29
GCL-IPL inferotemporal	79.0 (70.75-88.50)	81.5 (77.25-87.00)	0.32	76.5 (65.50-86.25)	79.0 (75.00-83.75)	0.39
GCL-IPL superotemporal	78.0 (69.00-84.50)	80.0 (75.00-85.00)	0.21	74.0 (64.75-83.75)	77.5 (71.50-84.75)	0.39

RNFL and GCL-IPL thickness according to gender

The median RNFL and GCL-IPL thickness values were generally higher in male patients compared to female patients in both eyes. In the right eye, the median RNFL thickness was 88.00 µm (IQR: 87.25-95.75 µm) in males and 88.35 µm (IQR: 88.35-95.25 µm) in females (p = 0.87). In the left eye, the median RNFL thickness was 92.00 µm (IQR: 87.75-97.50 µm) in males and 88.00 µm (IQR: 83.00-94.25 µm) in females (p = 0.13). A similar trend was observed in GCL-IPL thickness. In the right eye, the median GCL-IPL thickness was 85.50 µm (IQR: 82.00-89.75 µm) in males and 80.00 µm (IQR: 73.00-85.00 µm) in females (p = 0.07). In the left eye, it was 83.00 µm (IQR: 80.25-89.75 µm) in males and 78.00 µm (IQR: 73.00-83.25 µm) in females (p = 0.07).

Sectoral analysis of GCL-IPL thickness revealed statistically significant differences in certain regions. In the right eye, the inferotemporal (median: 86.00 µm; IQR: 82.50-89.75 µm in males vs. 79.50 µm; IQR: 72.75-87.00 µm in females, p = 0.02) and superotemporal sectors (84.00 µm; IQR: 82.25-91.75 µm vs. 78.00 µm; IQR: 72.75-84.00 µm, p = 0.01) showed significantly greater thickness in males. In the left eye, similar differences were observed in the inferotemporal (87.50 µm; IQR: 81.50-92.25 µm vs. 77.50 µm; IQR: 72.75-81.25 µm, p = 0.01), superotemporal (85.50 µm; IQR: 80.00-89.00 µm vs. 76.00 µm; IQR: 71.00-82.00 µm, p = 0.02), and inferior sectors (84.50 µm; IQR: 82.25-88.00 µm vs. 77.00 µm; IQR: 70.75-82.00 µm, p = 0.02). These findings suggest a consistent pattern of localized ganglion cell thinning in female patients, particularly in the inferotemporal and superotemporal regions. No significant intra-gender differences were observed between the right and left eyes (Table 3).

**Table 3:** Comparison of RNFL and GCL-IPL Thickness Between Males and Females (N = 50) RNFL: retinal nerve fiber layer; GCL-IPL: ganglion cell layer-inner plexiform layer; IQR: interquartile range; AVG: average; ^@^ male patients (n = 8); ^$^ female patients (n = 42); * p-value: comparison between males and females for each eye; ^@^ p-value: comparison between right and left eyes in males; ^$^ p-value: comparison between right and left eyes in females; ^#^ statistically significant difference (p < 0.05)

Nerve Parameter (Median; IQR)	Right Eye (Males)	Right Eye (Females)	*P-value	Left Eye (Males)	Left Eye (Females)	*P-value	^@^P-value	^$^P-value
AVG RNFL	88.00; 87.25-95.75	88.35; 88.35-95.25	0.87	92.00; 87.75-97.50	88.00; 83.00-94.25	0.13	0.44	0.77
AVG GCL-IPL	85.50; 82.00-89.75	80.00; 73.00-85.00	0.07	83.00; 80.25-89.75	78.00; 73.00-83.25	0.07	0.08	0.05
GCL-IPL superior	85.50; 82.75-88.75	81.50; 73.75-87.00	0.20	84.00; 73.25-91.00	81.00; 76.00-85.00	0.22	0.59	0.06
GCL-IPL superior nasal	87.50; 77.25-92.75	82.50; 77.00-88.00	0.24	83.50; 72.00-92.25	81.00; 76.00-85.00	0.55	0.23	0.11
GCL-IPL inferonasal	87.00; 81.00-92.75	81.00; 73.00-86.00	0.11	84.00; 77.00-91.00	80.00; 74.00-84.00	0.13	0.73	0.23
GCL-IPL inferior	86.00; 81.50-91.50	78.00; 72.75-84.25	0.06	84.50; 82.25-88.00	77.00; 70.75-82.00	0.02^#^	0.79	0.10
GCL-IPL inferotemporal	86.00; 82.50-89.75	79.50; 72.75-87.00	0.02^#^	87.50; 81.50-92.25	77.50; 72.75-81.25	0.01^#^	0.93	0.30
GCL-IPL superotemporal	84.00; 82.25-91.75	78.00; 72.75-84.00	0.01^#^	85.50; 80.00-89.00	76.00; 71.00-82.00	0.02^#^	0.61	0.16

Correlation of age with RNFL and GCL-IPL thickness

When analyzing the relationship between age and retinal parameters, average RNFL thickness showed a weak negative correlation with age in both eyes, but these findings were not statistically significant (right eye: r = -0.25, p = 0.07; left eye: r = -0.22, p = 0.12). In contrast, average GCL-IPL thickness exhibited a significant negative correlation with age. The correlation was stronger in the right eye (r = -0.43, p = 0.002) than in the left eye (r = -0.29, p = 0.03).

**Figure 1:**
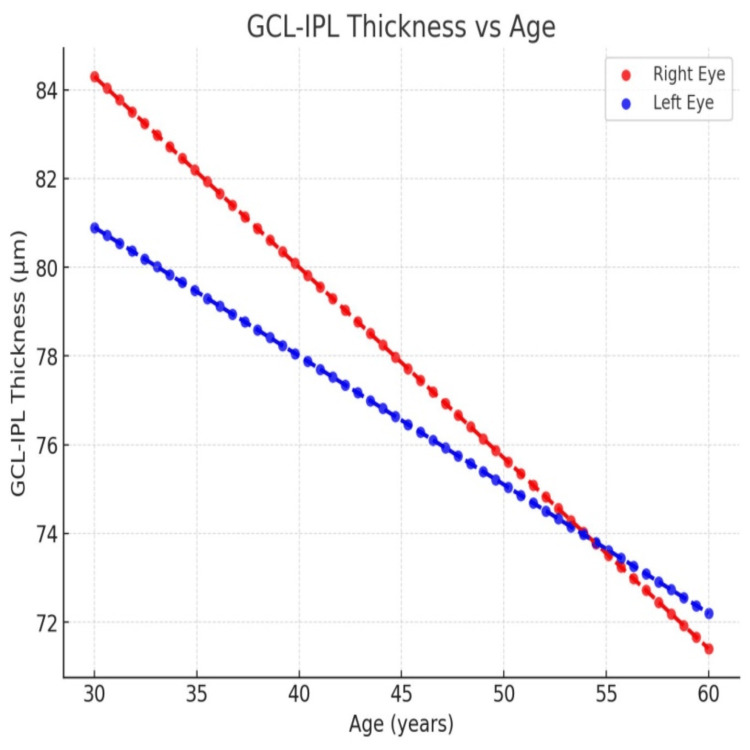
Scatter Plot of GCL-IPL Thickness Versus Age Showing Declining Trends in Both Eyes Red: right eye; Blue: left eye; GCL-IPL: ganglion cell layer-inner plexiform layer

Sectoral GCL-IPL analysis revealed significant age-related thinning in multiple regions. In the right eye, significant negative correlations were found in the superior (r = -0.29, p = 0.04), superior nasal (r = -0.43, p = 0.001), inferonasal (r = -0.50, p < 0.001), inferior (r = -0.48, p < 0.001), inferotemporal (r = -0.37, p = 0.008), and superotemporal (r = -0.29, p = 0.03) sectors. In the left eye, significant negative correlations were observed in the inferotemporal (r = -0.30, p = 0.03) and superotemporal (r = -0.33, p = 0.01) sectors. These findings indicate that age-related thinning is more pronounced in the GCL-IPL compared to the RNFL, with greater susceptibility in the inferotemporal and superotemporal regions (Table 4 and Figure 1).

**Table 4:** Correlation of Nerve Fiber Layer Thickness Parameters With Age in Hyperthyroid Patients Spearman correlation coefficients (ρ) are shown for each parameter in both eyes, with associated p-values indicating statistical significance. RNFL: retinal nerve fiber layer; GCL-IPL: ganglion cell layer-inner plexiform layer; AVG: average; P: probability value (statistical significance); # statistically significant (p < 0.05)

Nerve Parameter	Right Eye		Left Eye	
	Correlation coefficient^#^	P-value	Correlation coefficient^#^	P-value
AVG RNFL	-0.25	0.07	-0.22	0.12
AVG GCL-IPL	-0.43	0.002^#^	-0.29	0.03^#^
GCL-IPL superior	-0.29	0.04^#^	-0.27	0.05
GCL-IPL superior nasal	-0.43	0.001^#^	-0.25	0.07
GCL-IPL inferonasal	-0.50	0.00^#^	-0.18	0.18
GCL-IPL inferior	-0.48	0.00^#^	-0.26	0.06
GCL-IPL inferiotemporal	-0.37	0.008^#^	-0.30	0.03^#^
GCL-IPL superiotemporal	-0.29	0.03^#^	-0.33	0.01^#^

## Discussion

TED is an autoimmune condition associated with autoimmune thyroid disorders, with significant potential for vision-threatening complications [21]. DON remains the most severe complication of TED, though it occurs infrequently [22]. Even in the absence of clinical optic neuropathy, subclinical structural changes in the RNFL and GCL-IPL may be present. Although several differences did not reach statistical significance, these trends may still reflect clinically relevant subclinical changes that warrant further evaluation.

The current study provides valuable insight into early structural alterations in macular GCC and peripapillary RNFL among TED patients without clinical signs of optic neuropathy. Our findings support the growing recognition of SD-OCT as a crucial tool for detecting subtle optic nerve damage before visual function is affected. Notably, reductions in RNFL thickness were observed in patients with TED who also exhibited risk factors such as female gender, elevated IOP, and increasing age. These findings are consistent with earlier reports suggesting that TED-induced orbital pathology may lead to subtle but measurable optic nerve compromise. Such thinning is likely multifactorial, arising from mechanical compression due to orbital congestion, ischemia, and chronic inflammation. Our results are in agreement with Danesh-Meyer et al., who documented RNFL thinning secondary to compressive neuropathy in chiasmal lesions detected by OCT [17].

Changes in macular GCC thickness also support the hypothesis that TED affects retinal ganglion cells prior to the manifestation of clinical symptoms. As the ganglion cell layer is more directly affected in various optic neuropathies than the RNFL, GCC analysis has been increasingly recognized as a more sensitive early marker. In TED, inflammatory and immune-mediated mechanisms may contribute to early ganglion cell compromise, even before significant optic nerve compression occurs.

Stratified analysis by IOP, gender, and age further elucidated the pattern of RNFL and GCC thinning. A considerable subset of patients had elevated IOP, potentially contributing to increased mechanical stress on the optic nerve and further exacerbating structural damage. Ghenciu et al. have similarly shown that TED influences the progression and severity of open-angle glaucoma (OAG), with elevated IOP and optic nerve changes influenced by patient age and gender [23]. Our findings support these observations and underscore the importance of close monitoring in patients with raised IOP. Age-related thinning of both RNFL and GCL-IPL was observed, likely reflecting increased vulnerability of neural tissues with aging and cumulative exposure to orbital inflammation.

Although our cohort had a female predominance, consistent with the known epidemiology of TED, it is noteworthy that previous literature suggests males with TED often experience more severe disease and a higher risk of DON [24]. While our study did not identify statistically significant gender-based differences in average RNFL or GCL-IPL thickness, this may reflect the exclusion of patients with clinical DON, implying that early structural changes may occur uniformly across genders in subclinical stages.

The clinical implications of our findings are substantial. While TED is traditionally viewed as an orbital disorder, our results emphasize that it also induces early neurodegenerative changes. Recognizing these early changes is essential, as they may precede visual function loss. Timely identification of RNFL and GCC alterations could prompt earlier therapeutic intervention and possibly prevent irreversible optic nerve damage. Routine incorporation of OCT in the assessment of TED patients, regardless of overt optic neuropathy, is advisable. Identifying at-risk patients through OCT metrics may enable proactive measures such as corticosteroids, radiotherapy, or surgical decompression.

This study highlights the importance of SD-OCT in detecting early optic nerve involvement in TED. Even in the absence of DON, structural changes in RNFL and GCL-IPL are evident and clinically meaningful. Integrating OCT-based screening into the standard care of TED patients can aid in early diagnosis, guide timely interventions, and potentially improve visual outcomes. Future longitudinal studies with larger sample sizes, longer follow-up durations, and functional assessments such as visual field testing or electrophysiological analysis are warranted to validate the prognostic utility of RNFL and GCL-IPL measurements in TED.

Limitations of the study include its retrospective design and relatively small sample size, which may limit the generalizability of the findings. The relatively small sample size also reduces statistical power, and results should be interpreted with caution. Additionally, the exclusion of patients with clinical DON may have led to an underestimation of the true extent of optic nerve involvement. The absence of an age- and gender-matched control group limits our ability to attribute RNFL and GCL-IPL alterations exclusively to TED. A controlled comparative design is essential in future work. The study also lacked functional correlation through visual field testing or electrophysiological assessments, which could have provided a more comprehensive evaluation of optic nerve function. Other unassessed variables, including smoking status, thyroid hormone levels, treatment history, and ethnic background, may influence retinal findings and further limit the generalizability of our results. Future prospective studies with larger, more diverse populations and functional outcome measures are needed to confirm these observations.

## Conclusions

The findings from this study reinforce the clinical value of SD-OCT in monitoring patients with TED, even in the absence of overt DON. Early structural changes in RNFL and GCL-IPL can occur subclinically and may provide an opportunity for preemptive interventions. Regular OCT-based evaluation should be integrated into the routine follow-up of TED patients, particularly in aging individuals and those with elevated IOP, to ensure timely recognition of optic nerve compromise. Identifying subtle changes in these parameters can guide more personalized surveillance and therapeutic strategies aimed at preserving vision.

Overall, the findings suggest that while IOP alone may not significantly influence RNFL or GCL-IPL thickness in TED patients, age and gender contribute more prominently to structural alterations.TED patients with elevated IOP, increasing age, and female gender exhibited thinner RNFL and GCL-IPL, though most differences were not statistically significant. These findings highlight the role of OCT in detecting early, subclinical changes. However, our results require confirmation through larger, longitudinal studies with age- and gender-matched control groups to establish definitive associations.
